# Ectopic expression of a human cysteine aspartate-specific protease gene *caspase-3* induces cell death and affects the infection of tomato mosaic virus in *Nicotiana* plants

**DOI:** 10.5511/plantbiotechnology.25.1127a

**Published:** 2026-03-25

**Authors:** Yuki Fujii, Seishiro Kato, Kouta Kurihara, Yuta Yamagishi, Eriko Suzuki, Nobumitsu Sasaki, Yasuhiko Matsushita

**Affiliations:** 1 United Graduate School of Agricultural Science, Tokyo University of Agriculture and Technology, Fuchu, Tokyo 183-8509, Japan; 2 Graduate School of Agriculture, Tokyo University of Agriculture and Technology, Fuchu, Tokyo 183-8509, Japan; 3 Gene Research Center, Tokyo University of Agriculture and Technology, Fuchu, Tokyo 183-8509, Japan

**Keywords:** human caspase-3, hypersensitive reaction-like cell death, *Nicotiana benthamiana*, potato virus X, tomato mosaic virus

## Abstract

In animals, genes of the cysteine aspartate-specific protease (caspase) family play a crucial role in inducing cell death. Genes homologous to animal caspase genes have not been found in plants, and to date, there are no examples of the ectopic expression of animal caspase genes inducing cell death in plants. In this study, we investigated whether cell death could be induced by expressing the human *caspase-3* gene in plants. Wild-type *caspase-3* (*V266*), inactive type (*V266H*), and constitutively active type (*V266E*) genes were expressed in leaves of *Nicotiana benthamiana* and/or *Nicotiana tabacum* using two types of *Agrobacterium*-infiltration-type virus vectors and one type of mechanical inoculation-type virus vector. In all cases, cell death symptoms were induced by *V266* and *V266E* but not *V266H*. Similar results were obtained even when the *caspase-3* gene was split into coding regions for subunit 1 and subunit 2 and co-expressed. As for virus infection, expression of *V266* and *V266E* suppressed the infection of the tomato mosaic virus (ToMV). This suppression effect was particularly pronounced when the constitutively active type *V266E* was expressed using a virus vector of ToMV. With *V266E*, cell-to-cell movement of the virus was inhibited and long-distance movement did not occur. Furthermore, the expression of *V266* and *V266E* tended to increase the mRNA levels of defense-related genes in *N. benthamiana*. These results suggest that the expression of the human *caspase-3* gene induces cell death in plants, affects plant gene expression, and exerts an inhibitory effect against ToMV infection.

## Introduction

Programmed cell death (PCD) is an essential function of multicellular organisms that acts to efficiently eliminate their own cells in a variety of situations such as ontogeny, homeostasis, defense responses to pathogens, and suppression of cancer development (Newton et al. 2024; Sun and Peng 2009). In animals, cysteine aspartate-specific proteases (caspases) are involved in the induction of cell death, called apoptosis, a type of PCD (Fuchs and Steller 2011). The caspase family, composed of caspases, includes initiator and effector proteases, and during apoptosis induction, an activated initiator caspase (caspase-2, -8, -9, -10, -11, and -12) activates another set of effector caspase (caspase-3, -6, and -7), and the effector caspases cleave other proteins to perform apoptosis (Dho et al. 2025). All caspases are synthesized as an inactive form called pro-caspase, which undergoes dimerization/oligomerization followed by cleavage to become the active form (Julien and Wells 2017; Shi 2004).

In plants, PCD is induced in a variety of processes, including development and responses to biotic and abiotic stresses (Kacprzyk et al. 2024; Mukhtar et al. 2016; Valandro et al. 2020). Experiments with peptide substrates and inhibitors for animal caspases have shown that there is enzymatic activity in plants that is equivalent to that of animal caspases (del Pozo and Lam 1998; Ge et al. 2016; Korthout et al. 2000; Xu and Zhang 2009). Studies using the peptide inhibitors of caspases have reported that caspase-like activity is induced in defense responses against pathogens (Chichkova et al. 2004; Fernández et al. 2012; Hatsugai et al. 2004) and in cell death induction during development (Tran et al. 2014). However, no genes encoding proteins homologous to animal caspases have ever been found in plants (Bonneau et al. 2008).

*Bcl-2-associated X* (*Bax*), an apoptosis-associated gene in animals, is an example of an apoptosis-related gene that induces cell death when expressed in plants (Lacomme and Cruz 1999). However, there are no known examples of apoptosis-related animal genes belonging to the caspase family that can induce cell death when expressed in plants. Caspase-3 is an executor of apoptosis classified as an effector caspase in the caspase family (Ponder and Boise 2019; Porter and Jänicke 1999). In human caspase-3, the mutants V266H and V266E, in which the 266th valine of the pro-protein is replaced by histidine and glutamic acid, respectively, are known as inactive and constitutively active forms, respectively (Pop et al. 2003; Walters et al. 2009). We therefore examined whether the expression of wild-type (*V266*) and mutant (*V266H* and *V266E*) human *caspase-3* genes induces cell death when expressed in plants. In addition, we examined whether the expression of these genes suppresses plant virus infection and its effect on the expression of defense-related genes.

## Materials and methods

### Plant materials and plant growth conditions

*Nicotiana* plants (*Nicotiana tabacum* cv. Samsun NN, *N. tabacum* cv. Samsun nn and *N. benthamiana*) were grown at 25°C with a 16-h light/8-h dark photoperiod, following the approach in a previous study (Ogata et al. 2012). Plants at 5- to 8-weeks-old after sowing were utilized for further experiments.

### Construction of plasmids

The details of the construction of plasmids used in this study are described in the Supplementary materials and methods. Oligonucleotides for the plasmid constructions and the plasmids utilized in this study are listed in Supplementary Tables S1 and S2, respectively.

### *Agrobacterium* infiltration

Transformation, growth, and infiltration of *Agrobacterium tumefaciens* (syn. *Rhizobium radiobacter*) strain GV3101(pMP90) were performed following the approach in a previous study (Yoshikawa et al. 2025). Strain GV3101(pMP90) transformed with the pSoup helper plasmid (Hellens et al. 2000) was used for pGR107- and pGL-TocJ/TogJ-derived plasmids. After *Agrobacterium* infiltration, *N. tabacum* and *N. benthamiana* were kept at 20°C and 25°C, respectively. The ratio of the final OD_600_ value for the inoculum is summarized in Supplementary Table S3.

### Recombinant virus inoculation

Inoculation of recombinant viruses was performed essentially as described in a previous work (Ogata et al. 2012). Template plasmids pTogJ-Cas3, pTogJ-Cas3-V266H, and pTogJ-Cas3-V266E and the control plasmid pTocJ-GFP (Hori and Watanabe 2003) were digested with MluI and purified. One microgram of the purified DNAs was subjected to in vitro transcription in a volume of 25 µl with T7 RNA polymerase as described in an earlier work (Hori and Watanabe 2003). Leaves of *N. tabacum* cv. Samsun nn were inoculated mechanically by rubbing 8 µl of the transcripts using carborundum.

### Measurement of electrolyte leakage

Electrolyte leakage was measured essentially as described in an earlier work (Ogata et al. 2012). Leaf materials were excised from the injection site of the infiltrated plants and soaked in H_2_O at a ratio of 4 ml H_2_O per 1 cm^2^ of leaf materials. After incubation at room temperature for 6 h, conductivity of the water was measured using a conductivity meter (model B-173, Horiba, Kyoto, Japan) to determine initial conductivity. Then, samples were boiled to release all electrolytes and conductivity of the water was measured as final conductivity. The ratio of the initial to the final conductivity was expressed as %leakage. The analysis was performed against each biological replicate derived from independent experiments.

### Reverse transcription-quantitative PCR (RT-qPCR)

RT-qPCR was performed as described previously (Suzuki et al. 2024). Total RNA was isolated from tissue using the RNAiso plus reagent (Takara Bio, Shiga, Japan) according to the manufacturer’s instructions. cDNA was synthesized from total RNA with Oligo dT Primer and Random 6-mers using PrimeScript RT Master Mix (Takara Bio) or ReverTra Ace qPCR RT Master Mix (TOYOBO, Osaka, Japan). qPCR analysis was performed with the Thermal Cycler Dice Real Time System II MRX TP960 or System III TP970 (Takara Bio) using THUNDERBIRD Next SYBR qPCR Mix (TOYOBO). The ΔΔCt method was used to calculate relative mRNA or virus RNA levels (Livak and Schmittgen 2001). *NbEF1*α or *NbActin* cDNA was amplified as an internal control to normalize the mRNA or genome RNA level of each gene or virus. The analysis was performed against each biological replicate derived from independent experiments. The specific oligonucleotides used for amplification are listed in Supplementary Table S4.

### Fluorescence observation

Microscopic GFP fluorescence images were obtained using a BZ-X800 all-in-one fluorescence microscope (Keyence, Osaka, Japan) with a 4x objective lens and a filter set of GFP EX470/40 and DM495. The area of GFP fluorescence in the inoculated leaves was quantified using the ImageJ image-processing software (https://imagej.net/ij/ (Accessed Sep 26, 2024)). Images of GFP and chlorophyll fluorescence in the treated plant leaves were acquired under blue light (470 nm) using a Dual Head Ex Light System with a long-wave path filter (Biotools Inc., Gunma, Japan).

### Re-inoculation of leaf extracts

Cell extract was prepared from uninoculated upper leaves using a 50 mM potassium phosphate buffer solution (pH 7.0). The extract was then mechanically inoculated onto the leaves of *N. tabacum* cv. Samsun NN, which contains the *N* gene, and the plants were kept at 20°C. If the extract included the virus particles of tomato mosaic virus (ToMV), necrotic lesions would appear.

### SDS-polyacrylamide gel electrophoresis and Western blotting

Cell extract was prepared from leaf materials using a 10 mM or 50 mM potassium phosphate buffer solution (pH 7.0) and then subjected to SDS-polyacrylamide gel electrophoresis. The gel was subsequently stained with Coomassie Brilliant Blue R-250. Antiserum was raised in a rabbit using a synthetic polypeptide (NH_2_-NATQRVDDATV-COOH) corresponding to the amino acid sequence of the coat protein (CP) of tobacco mild green mosaic virus (TMGMV) (GenBank accession number: AB078435) between positions 110 and 120 (Eurofins Genomics, Tokyo, Japan). The relative position of the CP band in the stained SDS-PAGE gel was confirmed by Western blotting using rabbit antiserum to CP.

### Statistical analysis

Experimental data are represented as mean±SEM and were analyzed by Bartlett’s test and one-way ANOVA followed by Tukey–Kramer test or Steel–Dwass test where applicable. Statistical analyses were performed using Microsoft Excel and Statcel4 statistical software (OMS Publishing, Tokyo, Japan).

## Results

### Ectopic expression of *caspase-3 V266* and *V266E* induces cell death in plants

**Figure 1:**
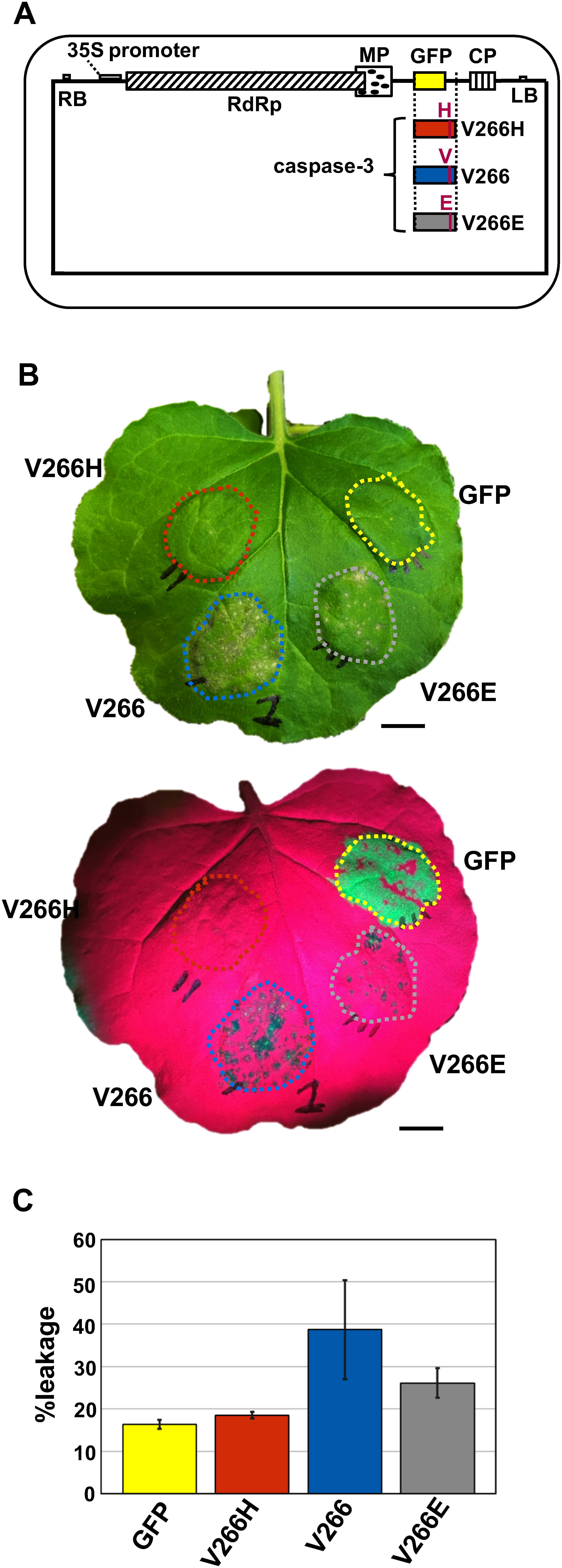
Figure 1. Cell death induction by *Agrobacterium*-infiltration-type ToMV vector-mediated gene expression of *caspase-3.* (A) Schematic drawing of the *Agrobacterium*-infiltration-type ToMV vector plasmids pGL-TocJ encoding the control GFP and pGL-TogJ encoding inactive-type caspase-3 (V266H), wild-type caspase-3 (V266) or constitutively active-type caspase-3 (V266E). (B) *Agrobacterium* containing the plasmid encoding GFP, V266H, V266, or V266E depicted in (A) were infiltrated into the leaves of *N. benthamiana* and the plants kept at 25°C. Turbidity of the *Agrobacterium* in each infiltration solution was OD_600_=0.3. Photographs were taken under white light (top) and blue light with a long-wave path filter (bottom) at 7 days after infiltration (dai). Dotted lines indicate each infiltrated area. Bar=1 cm. (C) Leaf materials at 7 dai in (B) were excised and soaked in water for 6 h. Conductivity of the water was measured before and after boiling the samples. The conductivity ratio was expressed as %leakage. Values are mean±SE (*n*=4). A nonparametric Steel–Dwass test was performed.

To examine whether ectopic expression of the animal *caspase-3* gene induces cell death in plants, we prepared human *caspase-3* cDNA (*V266*; wild-type, active form) and its mutants (*V266H*; inactive form and *V266E*; constitutively active form) and cloned them into various gene expression vectors (Supplementary materials and methods). First, we used the *Agrobacterium*-infiltration-type ToMV-based vector plasmids pGL-TocJ and pGL-TogJ to construct expression plasmids encoding control GFP or caspase-3 V266H, V266, or V266E (Figure 1A). pGL-TocJ and pGL-TogJ encode infectious cDNA clones of modified ToMV, which are composed of ToMV-derived replicase and movement protein genes and TMGMV-derived coat protein gene. Cell death symptoms were observed in the leaves of *N. benthamiana* infiltrated with *Agrobacterium* harboring the expression plasmid of active form V266 or constitutively active form V266E but not with those of inactive form V266H or control GFP (Figure 1B). Electrolyte leakage levels of V266 and V266E appeared slightly higher than those of V266H and control GFP (Figure 1C).

Virus vectors derived from ToMV and transcribed from the CaMV 35S promoter are known empirically to infect *N. benthamiana* at practical levels but not *N. tabacum* for unknown reasons. In fact, the *Agrobacterium*-infiltration-type virus vectors pGL-TocJ and pGL-TogJ were not suitable for the virus infection in *N. tabacum* (data not shown). Therefore, we utilized another *Agrobacterium*-infiltration-type virus vector pGR107, which was based on potexvirus and composed of potato virus X (PVX)-derived genes for a replicase, movement proteins (TGB1, TGB2 and TGB3) and a coat protein, to construct expression plasmids encoding control GFP or caspase-3 V266H, V266 or V266E (Supplementary Figure S1A). Cell death symptoms were observed in *N. tabacum* infiltrated with *Agrobacterium* harboring the expression plasmid of V266 or V266E but not with those of V266H or control GFP (Supplementary Figure S1B). The electrolyte leakage level of V266E was significantly higher than those of V266H, V266E, and control GFP (Supplementary Figure S1C). Similar results were obtained in *N. benthamiana* as well (Supplementary Figure S1D, E).

To examine the cell death-inducing ability of *caspase-3* *V266* and *V266E* under an *Agrobacterium*-free condition, we took advantage of mechanical inoculation type ToMV-based vectors pTocJ and pTogJ, in which the CaMV 35S promoter of the pGL-TocJ and pGL-TogJ vectors were respectively replaced by T7 RNA promoter for in vitro transcription, and constructed expression plasmids encoding control GFP or caspase-3 V266H, V266, or V266E (Supplementary Figure S2A). Leaves of *N. tabacum* were inoculated by rubbing the infectious virus RNAs transcribed in vitro from each expression plasmid. Necrotic lesions were observed in leaves inoculated with the in vitro transcribed RNAs of *V266* or *V266E*, but not in those of control *GFP* or *V266H* (Supplementary Figure S2B).

To examine whether *caspase-3 V266* and *V266E* induce cell death without employing virus vectors, cDNAs for V266H, V266 and V266E of caspase-3 and control sGFP were cloned into an *Agrobacterium*-infiltration-type vector, pART27-35Sa-GWB-DHA (Ogata et al. 2012), in which the gene of interest can be expressed under the control of CaMV 35S promoter. No symptoms of cell death were observed in *N. tabacum* and *N. benthamiana* following the infiltration of *Agrobacterium* containing these constructs (data not shown). It is known that active caspase-3 cleaves the VirD2 protein, which is essential for the gene transfer process in *Agrobacterium* (Chichkova et al. 2004). Leaky expression of *caspase-3* (*V266* and *V266E*) may occur, potentially inhibiting gene transfer efficiency due to cleavage of the VirD2 protein in *Agrobacterium*. Therefore, we planned to express the coding sequence of each subunit of caspase-3 (Supplementary Figure S3A). *Agrobacterium* harboring the expression plasmid encoding subunit 1 of caspase-3 was infiltrated into the leaves of *N. benthamiana* together with *Agrobacterium* containing one of three forms of subunit 2 of *caspase-3* (*V266H*, *V266*, and *V266E*) or control *GUS* (Supplementary Figure S3B). Cell death symptoms were observed when *V266* and *V266E* were used, but not when *V266H* and the control *GUS* were used (Supplementary Figure S3C). Furthermore, we investigated whether expressing subunits separately remains effective when one subunit is expressed using a virus vector. The coding sequence of caspase-3 subunit 1 was cloned into the above-mentioned *Agrobacterium*-infiltration-type virus vector pGR107 and expressed in *N. benthamiana* and *N. tabacum* together with the coding sequence of one of the three forms of subunit 2 of caspase-3 (*V266H*, *V266*, and *V266E*) (Supplementary Figure S3D). Cell death symptoms were observed in the areas treated with *V266* and *V266E*, but not with *V266H* in both *N. benthamiana* (Supplementary Figure S3E) and *N. tabacum* (Supplementary Figure S3F). The virus encoding caspase-3 subunit 1 does not encode erGFP, so successful infection of the virus cannot be observed via GFP fluorescence (Supplementary Figure S3D). Therefore, successful infection of the virus was indirectly monitored by using the control virus vector encoding erGFP and observing GFP fluorescence (Supplementary Figure S3E, F).

These results suggest that the ectopic expression of *caspase-3* *V266* and *V266E* induced cell death in *N. benthamiana* and *N. tabacum*.

### Ectopic expression of *caspase-3 V266* and *V266E* affects the infection of tomato mosaic virus

**Figure 2:**
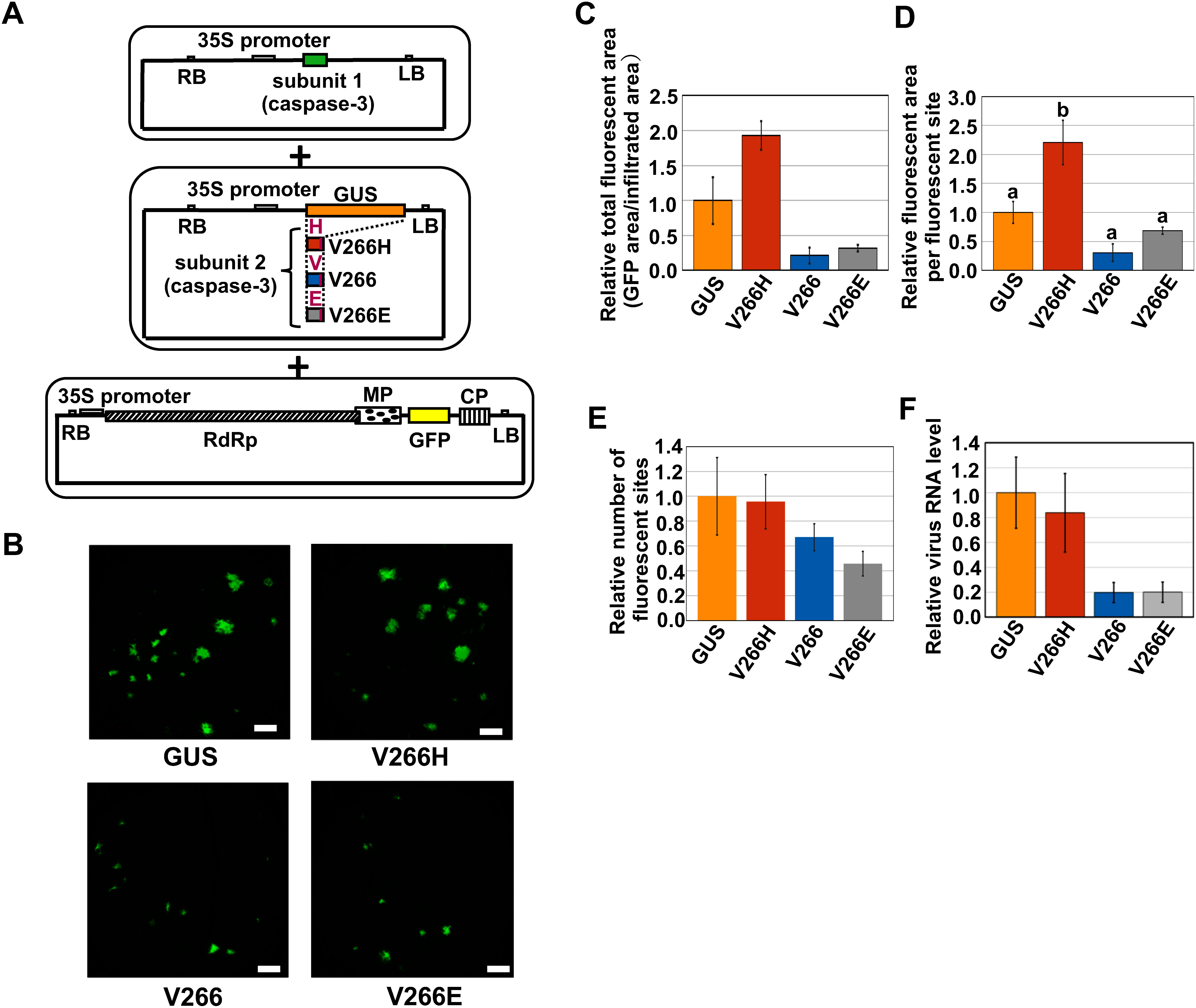
Figure 2. Effect of co-expression of the genes encoding subunit 1 and subunit 2 of caspase-3 on ToMV infection. (A) Schematic drawing of the plasmid constructs used for the co-infiltration of *Agrobacterium*. The first plasmid encodes subunit 1 of caspase-3, the second one encodes the control GUS or subunit 2 of the inactive-type (V266H), wild-type (V266), or constitutively active-type (V266E) of caspase-3, and the third one encodes the infectious cDNA clone of ToMV harboring GFP. (B) *Agrobacterium* containing the plasmid encoding subunit 1, one of GUS, V266H-, V266-, or V266E-type subunit 2 and the infectious cDNA clone of ToMV harboring GFP shown in (A) were co-infiltrated into the leaves of *N. benthamiana* and the plants kept at 25°C. Turbidity of the *Agrobacterium* mixture was OD_600_=0.18 and the OD_600_ values corresponding to each *Agrobacterium* from the first to third were 0.05, 0.05, and 0.08, respectively. Microscopic GFP fluorescence images at 3 days after infiltration are shown. Bar=1 mm. (C–E) The microscopic GFP fluorescence images in (B) were analyzed using ImageJ image-processing software to quantify the relative values of (C) the total fluorescent area per infiltrated area, (D) the fluorescent area per fluorescent site, and (E) the number of fluorescent sites by setting the means of the GUS control to 1. (F) The amount of virus RNA was analyzed by RT-qPCR using the total RNA isolated from the *Agrobacterium*-infiltrated leaves of the control GFP, V266H, V266 and V266E in (B) at 3 dai. Oligonucleotides for amplifying a part of the coding region for the virus RNA-dependent RNA polymerase were used for qPCR and the relative virus RNA levels were calculated by setting the means of the GUS control to 1. The virus RNA levels were normalized against the *NbEF1*α mRNA levels. Values are mean±SE (*n*=4). Data was analyzed by (C), (E), Steel–Dwass test and (D), (F), Tukey–Kramer test. Different letters above bars in (D) indicate statistically significant differences between means as a result of Tukey–Kramer test (*p*<0.05).

To examine whether the ectopic expression of *caspase-3* affects the infection of plant viruses, the infection of ToMV, in which its coat protein has been replaced by that of TMGMV, was analyzed in *N. benthamiana* leaves co-infiltrated with *Agrobacterium* harboring the plasmids producing subunit 1 of caspase-3, that producing one of three forms of subunit 2 (V266H, V266, and V266E) of caspase-3, and that producing infectious RNA of the ToMV encoding GFP (Figure 2A). *GUS* cDNA was used as a negative control. At 3 days after infiltration, the ratio of total GFP fluorescence area to total infiltrated leaf area seemed to be reduced by the infiltration of *V266* and *V266E* compared to *V266H* and control *GUS* (Figure 2B, C). Similarly, the size of GFP fluorescence area per fluorescent site seemed to be reduced by the treatment of *V266* and *V266E* (Figure 2D). These values seemed to increase more in the treatment with *V266H* compared to the *GUS* control (Figure 2C, D). On the other hand, the number of GFP fluorescent sites per total infiltrated leaf area seemed to decrease slightly by the treatment of *V266* and *V266E* compared to *V266H* and control *GUS* (Figure 2E). RT-qPCR revealed that the levels of ToMV RNA in *V266*- and *V266E*-expressing tissue were approximately 0.2-fold that of control *GUS*-expressing tissue, whereas the levels in *V266H*-expressing tissue were comparable to those in control *GUS*-expressing tissue (Figure 2F). These results suggest that the transient overexpression of cDNAs encoding subunit 1 and 2 (V266 and V266E, but not V266H) of caspase-3 affected the early stage of ToMV accumulation during infection.

To examine whether the ectopic expression of *caspase-3* has an inhibitory effect on virus infection even when it is synthesized only in virus-infected cells, we utilized the *Agrobacterium*-infiltration-type ToMV-based vectors pGL-TocJ and pGL-TogJ encoding the control GFP or one of three forms of caspase-3 (V266H, V266, and V266E) (Figure 1A). In this system, virus-encoding foreign genes are expressed only in virus-infected cells. At 6 days after infiltration, the levels of virus RNA in *V266E*-infiltrated leaves were less than 0.01-fold those of control *GFP*- or *V266H*-infiltrated leaves, whereas the levels in *V266*-infiltrated leaves were slightly lower compared to those in control *GFP*- or *V266H*-infiltrated leaves (Supplementary Figure S4A). SDS-PAGE analysis also revealed that the levels of coat proteins in *V266E*-infiltrated leaves were much lower than those of control *GFP*- or *V266H*-infiltrated leaves, whereas the levels in *V266*-infiltrated leaves were slightly lower compared to those in control *GFP*- or *V266H*-infiltrated leaves (Supplementary Figure S4B). These results suggest that the virus accumulation of ToMV was slightly reduced by the overexpression of *caspase-3 V266* and significantly reduced by *caspase-3 V266E* in virus-inoculated leaves.

Next, we examined the inhibitory effect of the ectopic expression of *caspase-3* on the long-distance movement of ToMV. *Agrobacterium* harboring pGL-TocJ and pGL-TogJ encoding the control GFP or one of the three forms of caspase-3 (V266H, V266, and V266E) (Figure 1A) were infiltrated into the leaves of *N. benthamiana*. At 16 days after infiltration, cell death symptoms were observed in the inoculated leaves of V266 and V266E but not V266H or the control GFP (Supplementary Figure S5A). In contrast, in the upper leaves, cell death symptoms were observed only in those of V266 but not V266E, V266H or the control GFP (Supplementary Figure S5A). To investigate whether virus particles exist in the upper leaves, the leaf extracts were inoculated by rubbing onto the leaves of *N* gene-containing *N. tabacum* cv. Samsun NN, in which necrotic lesions would appear due to the HR initiated by the interaction of *N*-gene product with the replicase of the ToMV if the leaf extracts include the virus particles. Necrotic lesions appeared in the leaves inoculated with the upper leaf extracts of the control GFP, V266H and V266 but not V266E (Supplementary Figure S5B). To further examine the existence of the virus particles in the upper leaves, those leaf extracts were also subjected to the SDS-PAGE to check the amount of coat protein of the virus. Bands representing coat proteins appeared in samples of V266, V266H and control GFP but barely appeared in V266E and not at all in uninfected controls (Supplementary Figure S5C). The reason a faint band of coat proteins was detected in the upper leaves in one of the samples of V266E (Supplementary Figure S5C lane V266E (P2)) seems to be that some deletion may have occurred in the coding region of caspase-3 V266E in the virus, since no necrotic legions were observed in the upper leaves (data not shown). Collectively, these results suggest that the long-distance movement of ToMV was suppressed by caspase-3 V266E but not the control GFP, V266H, and V266.

### Effect of ectopic expression of *caspase-3* on mRNA levels of defense-related genes

**Figure 3:**
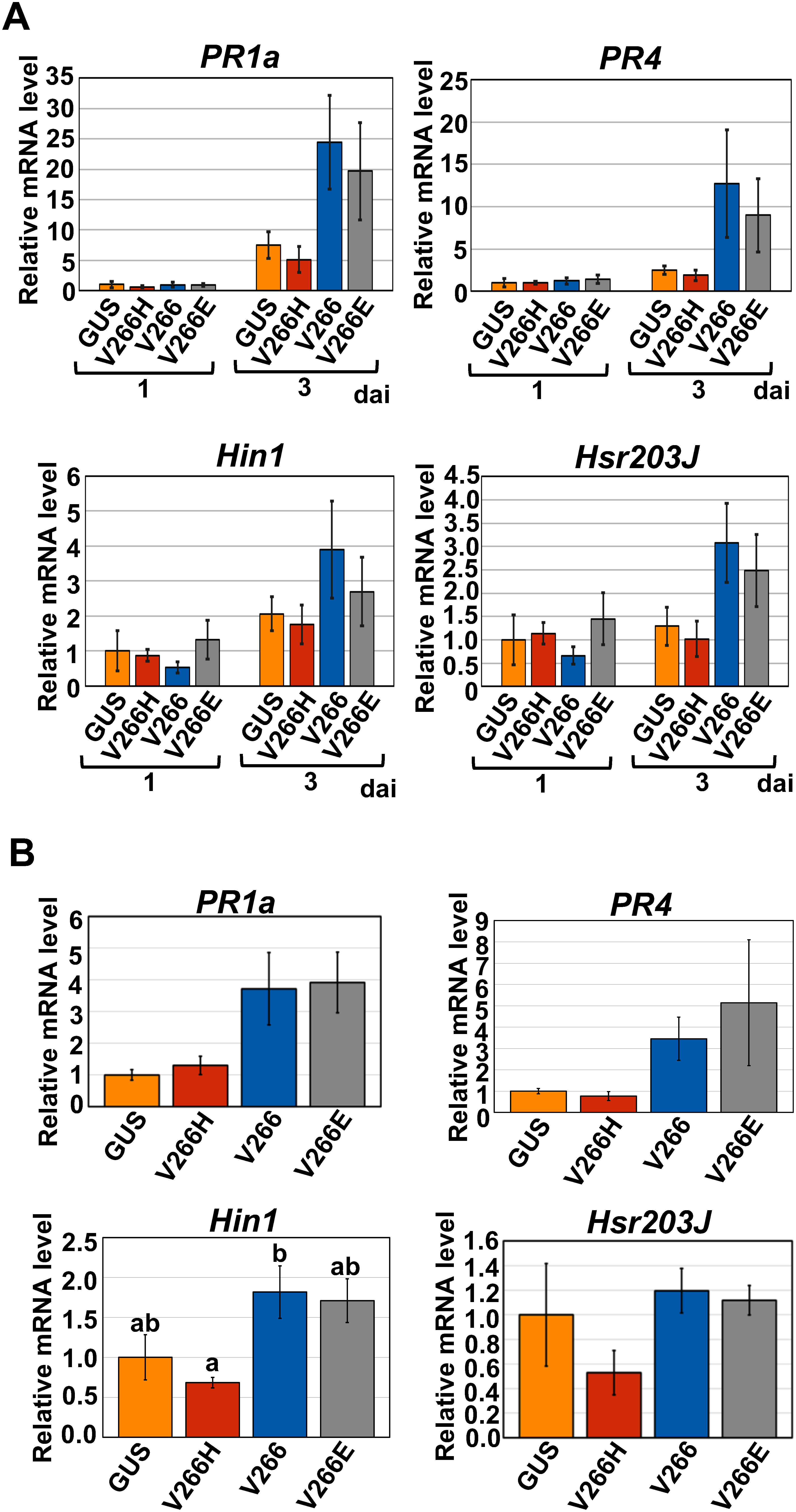
Figure 3. Effect of co-expression of the genes encoding subunit 1 and subunit 2 of caspase-3 on the mRNA levels of defense-related genes in the presence or absence of ToMV inoculation. *Agrobacterium* containing the plasmid encoding the subunit 1 and that encoding one of GUS, V266H-, V266-, or V266E-type subunit 2 were co-infiltrated into the leaves of *N. benthamiana* (A) without or (B) with that containing the plasmid for the infectious cDNA clone of GFP-encoding ToMV. In (A), the turbidity of the *Agrobacterium* mixture was OD_600_=0.2 and the OD_600_ values for each *Agrobacterium* were both 0.1. In (B), the turbidity of the *Agrobacterium* mixture was OD_600_=0.18 and the OD_600_ values for *Agrobacterium* containing each of the first to third plasmids were 0.05, 0.05, and 0.08, respectively. Infiltrated plants were kept at 25°C and the total RNAs were isolated from each infiltrated region at (A) 1 day (A), (B) and/or 3 days after infiltration (dai). RT-qPCR was performed using oligonucleotides for the defense-related genes *PR1*, *PR4*, *Hin1*, and *Hsr203J* of *N*. *benthamiana* to evaluate the relative mRNA levels of each gene. The mRNA levels of these target genes were normalized to that of *NbEF1*α, and the mRNA levels of the GUS control at (A) 1 dai and (B) 3 dai were fixed to 1 in each panel. Values are mean±SE (*n*=4). For statistical analyses, a Tukey–Kramer test was used as for *Hin1* in (B), and a Steel–Dwass test was used for the rest. Different letters above bars (as for *Hin1* in (B)) indicate statistically significant differences between the means revealed by the Tukey–Kramer test (*p*<0.05).

To determine whether the ectopic expression of the exogenous *caspase-3* gene affects the gene expression in plants, the mRNA levels of selected defense-related genes (i.e., *PR1a*, *PR4*, *Hin1*, and *Hsr203j*) were analyzed in *N. benthamiana*. *PR1a* and *PR4* were selected as marker genes for the salicylic acid and jasmonic acid signaling pathways, respectively, whereas *Hin1* and *Hsr203j* were used as resistance-related markers (Pascual et al. 2015; Yoshikawa et al. 2025). *N. benthamiana* leaves were co-infiltrated with *Agrobacterium* harboring the plasmids producing subunit 1 of caspase-3 and that producing one of three forms of subunit 2 (V266H, V266, and V266E) of caspase-3. *GUS* cDNA was utilized as a negative control. The mRNA levels of the four genes were comparable among the control GUS and the three forms of subunit 2 at 1 day after infiltration (dai) and seemed to increase by the infiltration of V266 and V266E compared to V266H and control GUS at 3 dai (Figure 3A). Similar results were obtained at 3 dai under the condition of ToMV infection (Figure 3B).

The mRNA levels of the four selected defense-related genes were also investigated by using the *Agrobacterium*-infiltration-type infectious clone of ToMV (Figure 1A) encoding the control GFP or one of three forms of caspase-3 (V266H, V266, and V266E). The mRNA levels of the four selected genes were comparable among the control GUS and the three forms of subunit 2 at 1 dai (Supplementary Figure S6). In contrast, at 4 dai, the mRNA levels of *PR1a* and *PR4* seemed to increase in V266 and V266E compared to V266H and control GUS, while those of *Hin1* and *Hsr203j* seemed to increase by V266 but not by others (Supplementary Figure S6). These results suggest that the mRNA levels of defense-related genes were upregulated by V266 and V266E of caspase-3.

## Discussion

In this study, we examined whether the expression of the human *caspase-3* gene in plants induces cell death through experiments utilizing multiple gene expression systems. In each analysis, instead of examining the presence of the produced caspase-3 protein by Western blot analysis or other methods, we utilized the coding sequences of three forms of caspase-3 (wild-type V266, constitutively active V266E, and inactive V266H) for comparison. For full-length caspase-3, the induction of cell death symptoms was confirmed by V266 and V266E but not V266H in *N. benthamiana* using the *Agrobacterium-*infiltration-type ToMV vector (Figure 1), in *N. benthamiana* and *N. tabacum* using the *Agrobacterium-*infiltration-type PVX vector (Supplementary Figure S1), and in *N. tabacum* using the mechanical inoculation-type ToMV vector of in vitro transcripts (Supplementary Figure S2). For the co-production of each subunit of caspase-3 (Supplementary Figure S3A), induction of cell death symptoms was also confirmed by V266 and V266E but not V266H in *N. benthamiana* utilizing the direct gene expression type vector by the CaMV 35S promoter (Supplementary Figure S3B, C) and in *N. benthamiana* and *N. tabacum* using the *Agrobacterium-*infiltration-type PVX vector for subunit 1 and the direct gene expression type vector for subunit 2 (Supplementary Figure S3D, E, F). Since induction of cell death symptoms was observed by V266 and V266E but not V266H or the negative controls GFP and GUS in all cases, we can conclude that the ectopic expression of the active-type human *caspase-3* genes of V266 and V266E induces cell death in plants.

In animals, caspase-3 is synthesized as a pro-type without activity and converted into the active form by activated initiator caspases such as caspase-9 (Julien and Wells 2017; Shi 2004). Therefore, our finding that the expression of the gene for wild-type human caspase-3 (*V266*) resulted in the induction of cell death in plants (Figure 1, Supplementary Figures S1, S2, S3, S5) suggests that an enzyme with an activity equivalent to that of caspase-9 in animals was present in plants and acted on the pro-type of caspase-3. It also means that caspase-3 acted on a plant protein to induce cell death in plants, although it is not known which protein caspase-3 targets in plants. It will be interesting to focus on identifying plant enzymes that activate wild-type human caspase-3 and the target proteins of caspase-3 that induce cell death in future work. Moreover, our finding that the mRNA levels of genes known to vary in expression during the cell death-associated HR, one of the plant defense responses to pathogens, were altered by the expression of the *caspase-3 V266* and *V266E* (Figure 3, Supplementary Figure S6) could be interpreted as indicating that *caspase-3* acted on the plant’s intrinsic signaling pathway for cell death induction. This is intriguing in that animal genes lacking the homologous genes in plants might be utilized to elucidate signaling pathways in plants.

Necrotic lesions extended beyond the *Agrobacterium*-infiltrated area and were observed throughout the leaf in the inoculated leaves of V266 (Supplementary Figure S5A), suggesting that the expression of *caspase-3 V266* induced cell death but did not stop cell-to-cell movement of the virus, and then induced cell death in the spread cells as well. As for V266, cell death symptoms appeared even in the upper leaves that were not inoculated with the virus (Supplementary Figure S5A). This may have been because the virus was not contained in the inoculated leaf when V266 was synthesized but moved to the upper leaves, where it replicated and synthesized V266 and thereby induced cell death. In contrast, for V266E, cell death symptoms did not appear (Supplementary Figure S5A), meaning that the virus encoding caspase-3 V266E did not move to the upper leaves. In the inoculated leaves, both V266- and V266E-encoding viruses induced cell death, but while necrotic lesions continued to expand for V266, they did not expand for V266E (Supplementary Figure S5A). It is known that V266 is not active unless the produced pro-form is processed, while V266E has protease activity even in its unprocessed pro-form (Pop et al. 2003; Walters et al. 2009). This difference between V266 and V266E may affect the timing of cell death induction and explain the difference in whether or not the cell-to-cell and long-distance movement of the virus can be suppressed. When tobacco mosaic virus (TMV) is inoculated to the leaves of *N* gene-carrying tobacco, HR is induced, necrotic lesions appear, the virus is contained in and around the necrotic lesions and virus transfer to the upper leaves does not occur. In contrast, when TMV is inoculated to the leaves of transgenic *N* gene-carrying tobacco expressing the gene for the baculovirus-derived apoptosis inhibitor protein IAP, cell death induction is delayed, the expansion of the necrotic lesions continues and the virus spreads to the upper leaves (del Pozo and Lam 2003). It is interesting to note that differences in the timing of cell death induction may affect whether or not virus containment occurs.

In this paper, while we investigated whether ectopic expression of *caspase-3* affects infection with ToMV, a tobamovirus (Figure 2, Supplementary Figures S4, S5), we did not address whether it affects infection with PVX, a potexvirus. Since we have prepared *Agrobacterium-*infiltration-type PVX vectors encoding each type of caspase-3 and confirmed cell death induction by V266 and V266E (Supplementary Figures S1, S3D, E, F), it remains to analyze the effect of *caspase-3* expression on the proliferation of PVX.

The effects of V266 and V266E on the mRNA levels of defense-related genes shown in Figure 3 were comparable, whereas the effects of V266E were somewhat weaker for *PR4* and *Hsr203J* than those of V266 in Supplementary Figure S6. In the experimental system in Figure 3, each subunit of caspase-3 was co-produced in cells directly from the mRNAs transcribed from the CaMV 35S promoter. In contrast, in the experimental system in Supplementary Figure S6, a ToMV-derived virus vector was utilized and caspase-3 was produced after the subgenomic RNA for caspase-3 was transcribed in cells where the virus was replicating. It is possible that the differing effects of V266 and V266E on the RNA levels of defense-related genes (Supplementary Figure S6) were related to their differing effect on the cell-to-cell and long-distance movement of the virus (Supplementary Figure S5).

This study is the first to report that cell death is induced by the expression in plants of a gene of the animal caspase family, which has no homologous gene in plants. It remains to be addressed what plant factors act in plants to activate caspase-3 and which proteins in plants are targeted by the activated caspase-3 for cell death induction and virus containment. Investigating the expression changes of plant genes when caspase-3 acts in plants is expected to deepen our understanding of the molecular mechanisms underlying cell death induction and pathogen containment in plants. Furthermore, the ability of animal caspase genes to induce cell death and contain pathogens in plants can be viewed as a useful tool for plant biotechnology, suggesting potential applications in future practical uses.

## Abbreviations

Bax: Bcl-2-associated X
CaMV: cauliflower mosaic virus
caspase: cysteine aspartate-specific protease
erGFP: endoplasmic reticulum-targeted GFP
PCD: programmed cell death
PVX: potato virus X
RT-qPCR: reverse transcription-quantitative PCR
TMGMV: tobacco mild green mosaic virus
TMV: tobacco mosaic virus
ToMV: tomato mosaic virus

## Accession numbers

The sequence data for the human *caspase-3* gene utilized in this study can be found in the International Nucleotide Sequence Database (INSD; GenBank/EMBL/DDBJ) under the INSD accession number BC016926.

## Description of supplementary files

Supplementary materials and methods. Construction of plasmids.

Supplementary Figure S1. Cell death induction by *Agrobacterium*-infiltration-type PVX vector-mediated gene expression of *caspase-3.*

Supplementary Figure S2. Cell death induction by mechanical inoculation-type ToMV vector-mediated gene expression of *caspase-3.*

Supplementary Figure S3. Cell death induction by co-expression of genes encoding subunit 1 and subunit 2 of caspase-3.

Supplementary Figure S4. Decreased accumulation of caspase-3-encoding ToMV in inoculated leaves.

Supplementary Figure S5. Cell death symptoms and virus accumulation in non-inoculated upper leaves of *N. benthamiana* inoculated by caspase-3-encoding ToMV.

Supplementary Figure S6. Transcript levels of defense-related genes in the leaves expressing *caspase-3* by *Agrobacterium*-infiltration-type ToMV vector.

Supplementary Table S1. Oligonucleotides used for plasmid construction.

Supplementary Table S2. Plasmids used in this study.

Supplementary Table S3. Turbidity of bacteria used for *Agrobacterium*-infiltration.

Supplementary Table S4. Oligonucleotides used in RT-qPCR analysis.
