# Supplementary material for: Ectopic expression of a human cysteine aspartate-specific protease gene caspase-3 induces cell death and affects the infection of tomato mosaic virus in Nicotiana plants
Source: Plant Biotechnol (Tokyo). 2026 Mar 25;43(1):95–103. doi: 10.5511/plantbiotechnology.25.1127a (PMC13170818; doi:10.5511/plantbiotechnology.25.1127a)
Supplement: Supplementary Data [file plantbiotechnology-43-1-25.1127a-s001.pdf]

Supplementary materials and methods

Construction of plasmids

The oligonucleotides used for plasmid construction and the plasmids used in this study are listed in Supplementary Tables S1 and S2, respectively.

1. Cloning of human *caspase-3* gene

Total RNA was isolated from the cisplatin-treated HepG2 cells using the RNAiso plus reagent (Takara Bio, Shiga, Japan) according to the manufacturer's instructions. cDNA was synthesized from total RNA with Oligo dT Primer and Random 6-mers using PrimeScript RT Master Mix (Takara Bio) and subjected to PCR with a set of Caspase3-human-F01 and Caspase3-human-R01 oligonucleotides to amplify the cDNA for human *caspase-3*. The resultant DNA fragment was cloned into the vector plasmid pGEM-T Easy (Promega, Madison, WI, USA) to construct the pGEM-Cas3 plasmid. The nucleotide sequence of the cloned cDNA was confirmed to be 100% identical to the coding sequence of human *caspase-3* (accession number: BC016926) in the International Nucleotide Sequence Database (INSD; GenBank/EMBL/DDBJ).

2. Construction of entry plasmids

The pGEM-Cas3 plasmid was utilized as a template for PCR with a set of Caspase3-human-F01 and Caspase3-human-R02 oligonucleotides and the resultant DNA was cloned into the vector plasmid pENTR-D/TOPO (Thermo Fisher Scientific, Waltham, MA, USA) to construct the entry plasmid pENTR-Cas3.

To construct the plasmid encoding the mutant type of caspase-3 V266E, pGEM-Cas3 plasmid was used as a template for PCR with a set of Caspase3-human-F01 and Caspase3-human-R03 oligonucleotides and the amplified fragment was cloned into pENTR-D/TOPO to construct the plasmid pENTR-Cas3-V266E_no_stop_codon. Then, the pENTR-Cas3-V266E_no_stop_codon plasmid was used as a template for inverse PCR with a set of pENTR-F01 and Caspase3-human-R02 oligonucleotides. The amplified fragment was phosphorylated and self-ligated to construct the entry plasmid pENTR-Cas3-V266E, in which the TAA stop codon was inserted following the coding sequence of caspase-3 V266E.

To construct the plasmid encoding the mutant type of caspase-3 V266H, pENTR-Cas3-V266E_no_stop_codon plasmid was used as a template for inverse PCR with a set of pENTR-F01 and Caspase3-human-R04 oligonucleotides. The amplified fragment was phosphorylated and self-ligated to construct the entry plasmid pENTR-Cas3-V266H.

To construct the entry plasmid for subunit 1 of caspase-3, pENTR-Cas3 plasmid was used as a template for inverse PCR with a set of pENTR-F02 and Cas3-Sub1-R01 oligonucleotides, and the amplified fragment was phosphorylated and self-ligated to construct the plasmid pENTR-Cas3-sub1_with_stop_codon_first_stage, which included the coding sequence of subunit 1 as well as the pre-sequence of caspase-3. To remove the pre-sequence of caspase-3, the pENTR-Cas3-sub1_with_stop_codon_first_stage plasmid was used as a template for inverse PCR with a set of Cas3-Sub1-F01 and pENTR-R02 oligonucleotides and the amplified fragment was phosphorylated and self-ligated to construct the entry plasmid pENTR-Cas3-sub1_with_stop_codon.

To construct the entry plasmids for the wild- and mutant-type subunit 2 of caspase-3, pENTR-Cas3, pENTR-Cas3-V266E and pENTR-Cas3-V266H plasmids were utilized as templates for inverse PCR with a set of Cas3-Sub2-F02 and pENTR-R02 oligonucleotides and the amplified fragments were phosphorylated and self-ligated to construct the entry plasmids pENTR-Cas3-sub2_with_stop_codon, pENTR-Cas3-sub2-V266E_with_stop_codon and pENTR-Cas3-sub2-V266H_with_stop_codon, respectively.

3. *Agrobacterium*-infiltration-type plasmid for direct gene expression by the CaMV 35S

promoter (e.g., pART27-35Sa-Cas3)

To construct the direct gene expression plasmids for the full-length caspase-3 and its mutant derivatives (V266E and V266H), entry plasmids pENTR-Cas3, pENTR-Cas3-V266E and pENTR-Cas3-V266H were subjected to *in vitro* recombination with the vector plasmid pART27-35Sa-GWB-DHA (Ogata et al. 2012) by using LR clonase II (Thermo Fisher Scientific) to construct the expression plasmids pART27-35Sa-Cas3, pART27-35Sa-Cas3-V266E and pART27-35Sa-Cas3-V266H, respectively.

To construct the direct gene expression plasmid for subunit 1 of caspase-3, entry plasmid pENTR-Cas3-sub1_with_stop_codon was subjected to the *in vitro* LR recombination with the vector plasmid pART27-35Sa-GWB-DHA to construct the expression plasmid pART27-35Sa-Cas3-sub1.

To construct the direct gene expression plasmids for subunit 2 of caspase-3, entry plasmids pENTR-Cas3-sub2_with_stop_codon, pENTR-Cas3-sub2-V266E_with_stop_codon and pENTR-Cas3-sub2-V266H_with_stop_codon were subjected to the *in vitro* LR recombination with the vector plasmid pART27-35Sa-GWB-DHA to construct the expression plasmids pART27-35Sa-Cas3-sub2, pART27-35Sa-Cas3-sub2-V266E and pART27-35Sa-Cas3-sub2-V266H, respectively.

The control plasmid pART27-35Sa-GUS was as described previously (Suzuki et al. 2024).

4. *Agrobacterium*-infiltration-type PVX plasmid (e.g., pGR107-Cas3)

4-1. Construction of modified PVX vector

Plasmid pGR107-XV_no_MluI_site_with_TAA_stop_codon was constructed using the following procedure to serve as a useful plasmid after SmaI digestion for cloning target genes via *in vitro* recombination using the SLiCE method (Motohashi 2015). The DNA fragment containing the coding region of an artificial transcription factor XVE was amplified from the pMDC150 plasmid (Brand et al. 2006) by PCR using a set of XVE-F01 and XVE-R02-w/o-stop-codon oligonucleotides. The amplified fragment was cloned into pENTR-D/TOPO to construct the plasmid pENTR-XVE_no_stop_codon. The resultant pENTR-XVE_no_stop_codon plasmid was used as a template for inverse PCR with a set of pENTR-F02 and XVE-V-R01 oligonucleotides, and the amplified fragment was phosphorylated and self-ligated to construct the plasmid pENTR-XV_with_TAA_stop_codon. Then, the pENTR-XV_with_TAA_stop_codon plasmid was utilized as a template for the second inverse PCR with a set of XV-F01 and XV-R01 oligonucleotides. The amplified fragment was phosphorylated and self-ligated to construct the plasmid pENTR-XV_no_MluI_site_with_TAA_stop_codon, in which the MluI site in the XV-coding region was removed by changing the nucleotide sequence. The resultant pENTR-XV_no_MluI_site_with_TAA_stop_codon plasmid was used for *in vitro* recombination with the vector plasmid pTogJ by using LR clonase II (Thermo Fisher Scientific) to construct the plasmid pTogJ-XV_no_MluI_site_with_TAA_stop_codon. Then, the pTogJ-XV_no_MluI_site_with_TAA_stop_codon plasmid was used as a template for PCR with a set of XV-pGR107 F01 and XV-pGR107-R02 oligonucleotides, and the amplified fragment was subjected to the *in vitro* SLiCE recombination with the SmaI- and SalI-digested pGR107-erGFP (Suzuki et al. 2024) to construct the plasmid pGR107-XV_no_MluI_site_with_TAA_stop_codon.

4-2. Construction of the PVX plasmids encoding caspase-3

To construct the *Agrobacterium*-infiltration-type PVX plasmids encoding the full length caspase-3 and its mutant derivatives (V266E and V266H), pART27-35Sa-Cas3, pART27-35Sa-Cas3-V266E and pART27-35Sa-Cas3-V266H plasmids were utilized as templates for PCR with a set of hCas3-pGR107-F01 and hCas3-pGR107-R01 oligonucleotides, and the amplified fragments were used for the *in vitro* SLiCE recombination with the SmaI-treated pGR107-XV_no_MluI_site_with_TAA_stop_codon to construct the plasmids pGR107-Cas3, pGR107-

Cas3-V266E and pGR107-Cas-V266H, respectively.

To construct the *Agrobacterium*-infiltration-type PVX plasmids encoding subunit 1 of caspase-3, pART27-35Sa-Cas3 plasmid was utilized as a template for PCR with a set of hCas3Sub1-pGR107-F01 and hCas3Sub1-pGR107-R01 oligonucleotides. The amplified fragment was utilized for *in vitro* SLiCE recombination with the SmaI-digested pGR107-XV_no_MluⅠ_site_with_TAA_stop_codon to construct the plasmid pGR107-Cas3-sub1.

The control plasmid pGR107-erGFP was as described previously (Suzuki et al. 2024).

5. Mechanical inoculation-type ToMV plasmids (e.g., pTogJ-Cas3)

Entry plasmids pENTR-Cas3, pENTR-Cas3-V266E and pENTR-Cas3-V266H were subjected to the *in vitro* LR recombination with the vector plasmid pTogJ (Hori and Watanabe 2003) to construct the plasmids pTogJ-Cas3, pTogJ-Cas3-V266E and pTogJ-Cas3-V266H, respectively.

The control plasmid pTocJ-GFP was as described previously (Hori and Watanabe 2003).

6. *Agrobacterium*-infiltration-type ToMV plasmid (e.g., pGL-TogJ-Cas3)

The plasmids pTogJ-Cas3, pTogJ-Cas3-V266E and pTogJ-Cas3V-266H were used as templates for PCR with a set of oligonucleotides TogJ-seq-F10-2 and TogJ-pGLW3-R01 and the amplified fragments were subjected to the *in vitro* SLiCE recombination with the MluI- and AatII-digested pGLW3 (Sasaki et al. 2013) to construct the plasmids pGL-TogJ-Cas3, pGL-TogJ-Cas3-V266E and pGL-TogJ-Cas3 V266H, respectively.

The plasmids pTocJ-GFP was used as a template for PCR with a set of oligonucleotides TogJ-seq-F10-2 and TogJ-R04 and the amplified fragment was subjected to the *in vitro* SLiCE recombination with the MluI- and AatII-digested pGLW3 (Sasaki et al. 2013) to construct the plasmids pGL-TocJ-GFP.

References

Brand L, Hörler M, Nüesch E, Vassalli S, Barrell P, Yang W, Jefferson RA, Grossniklaus U, Curtis MD (2006) A versatile and reliable two-component system for tissue-specific gene induction in Arabidopsis. *Plant Physiol* 141: 1194–1204

Hori K, Watanabe Y (2003) Construction of a tobamovirus vector that can systemically spread and express foreign gene products in solanaceous plants. *Plant Biotechnol* 20:129–136

Motohashi K (2015) A simple and efficient seamless DNA cloning method using SLiCE from *Escherichia coli* laboratory strains and its application to SLiP site-directed mutagenesis. *BMC Biotechnol* 15: 47

Ogata T, Kida Y, Arai T, Kishi Y, Manago Y, Murai M, Matsushita Y (2012) Overexpression of tobacco ethylene response factor *NtERF3* gene and its homologues from tobacco and rice induces hypersensitive response-like cell death in tobacco. *J Gen Plant Pathol* 78: 8–17

Sasaki N, Takaoka M, Sasaki S, Hirai K, Meshi T, Nyunoya H (2013) The splice variant Ntr encoded by the tobacco resistance gene *N* has a role for negative regulation of antiviral defense responses. *Physiol Mol Plant Pathol* 84: 92–98

Suzuki H, Ito T, Ogata T, Tsukahara Y, Nelson RS, Sasaki N, Matsushita Y (2024) Overexpression of *NtERF5*, belonging to the ethylene response factor gene family, inhibits potato virus X infection and enhances expression of jasmonic acid/ethylene signaling marker genes in tobacco. *J Gen Plant Pathol* 90: 125–133

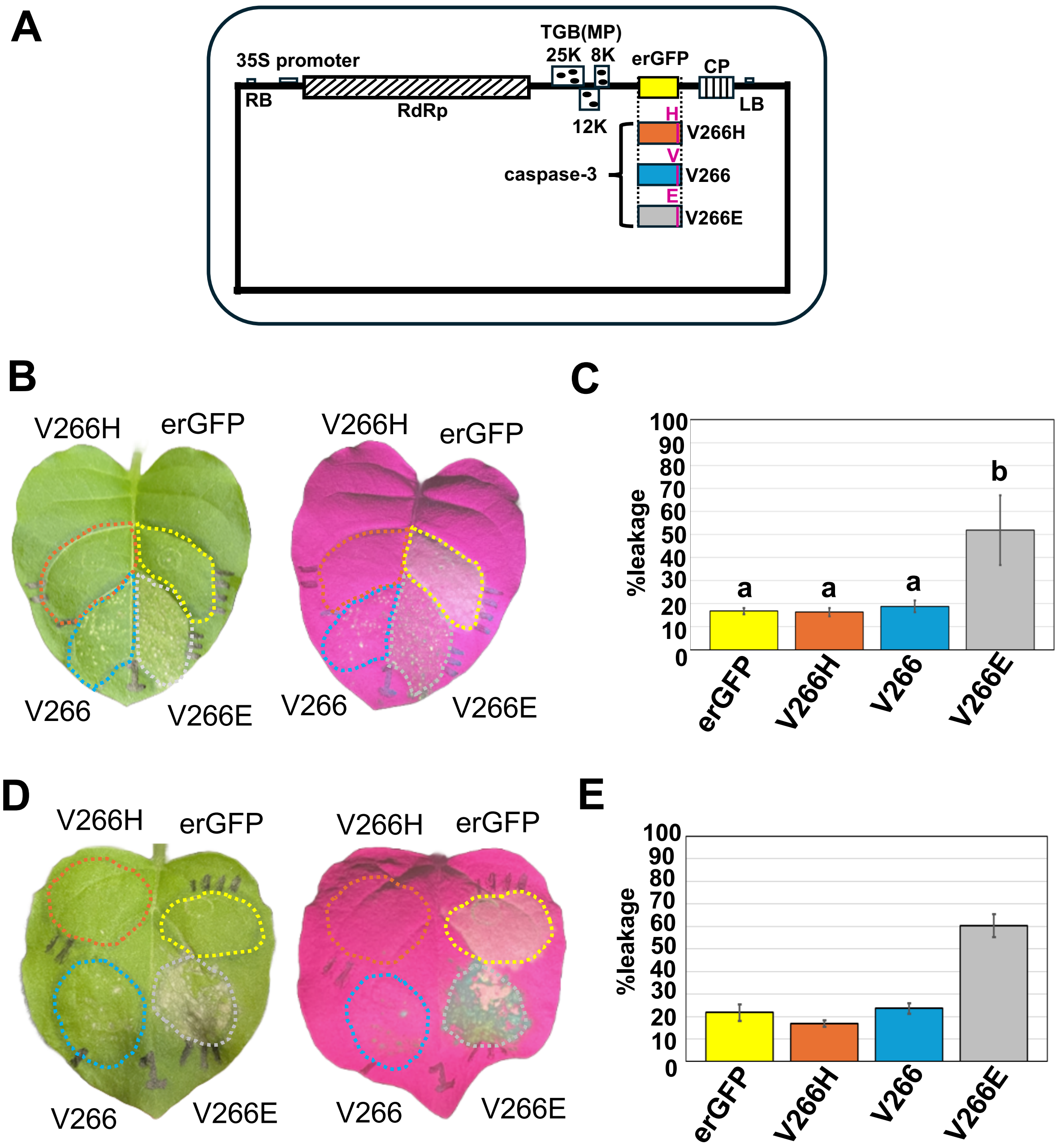

Supplementary Figure S1. Cell death induction by *Agrobacterium*-infiltration-type PVX vector-mediated gene expression of *caspase-3*.

(A) Schematic drawing of the *Agrobacterium*-infiltration-type PVX vector plasmid pGR107 encoding the control erGFP and inactive-type caspase-3 (V266H), wild-type caspase-3 (V266), or constitutively active-type caspase-3 (V266E). (B), (D) *Agrobacterium* containing the plasmid encoding GFP, V266H, V266, or V266E shown in (A) were infiltrated into the leaves of (B) *N. tabacum* cv. Samsun nn and (D) *N. benthamiana* and the plants kept at (B) 20°C or (D) 25°C, respectively. The turbidity of *Agrobacterium* in each infiltration solution was OD_{600} = 0.01. Photographs were taken under white light (left) and blue light with a long-wave path filter (right) at (B) 8 or (D) 6 days after infiltration (dai). Dotted lines indicate each infiltrated area. (C), (E) Leaf materials at 8 dai in (B) and 6 dai in (D) were excised and soaked in water for 6 h. Conductivity of the water was measured before and after boiling the samples. The conductivity ratio was expressed as %leakage. Values are mean ± SE (n = 4). For statistical analyses, Tukey–Kramer test and Steel–Dwass test were used for (C) and (E), respectively. Different letters above bars in (C) indicate significant differences as a result of Tukey–Kramer test ($p < 0.05$).

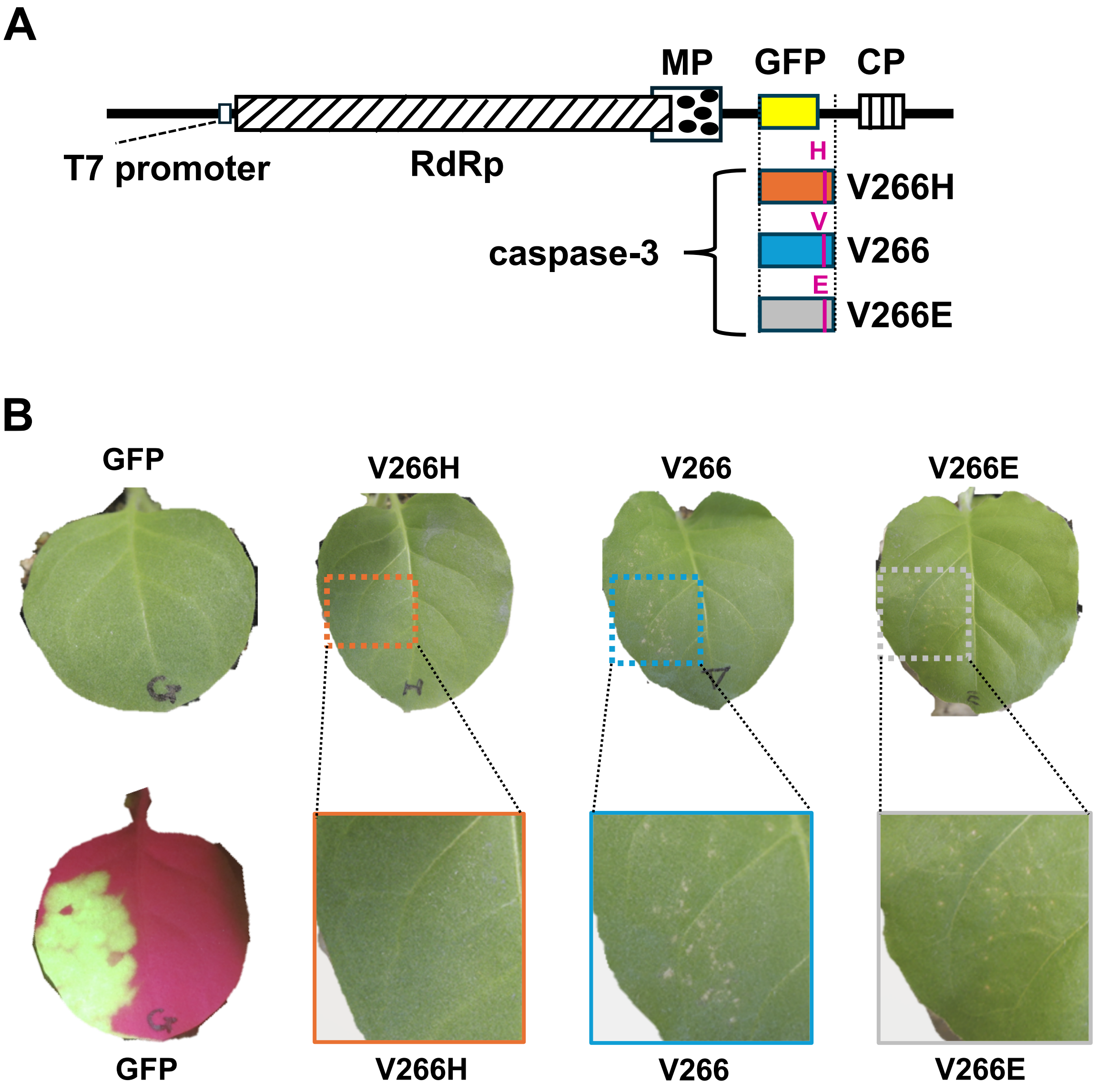

Supplementary Figure S2. Cell death induction by mechanical inoculation-type ToMV vector-mediated gene expression of *caspase-3*.

(A) Schematic drawing of the mechanical inoculation-type ToMV vector plasmids pTocJ encoding the control GFP and pTogJ encoding inactive-type caspase-3 (V266H), wild-type caspase-3 (V266), or constitutively active-type caspase-3 (V266E). (B) The virus genome RNAs transcribed *in vitro* from the plasmids encoding GFP, V266H, V266, or V266E shown in (A) were subjected to the mechanical inoculation onto the left half of *N. tabacum* cv. Samsun nn leaves and the plants kept at 20˚C. Photographs were taken under white light (top) and blue light with a long-wave path filter (lower left, GFP) at 7 days after infiltration. Enlarged views of the photo sections outlined in dotted squares are shown in the lower right (V266H, V266, V266E).

Supplementary Figure S3

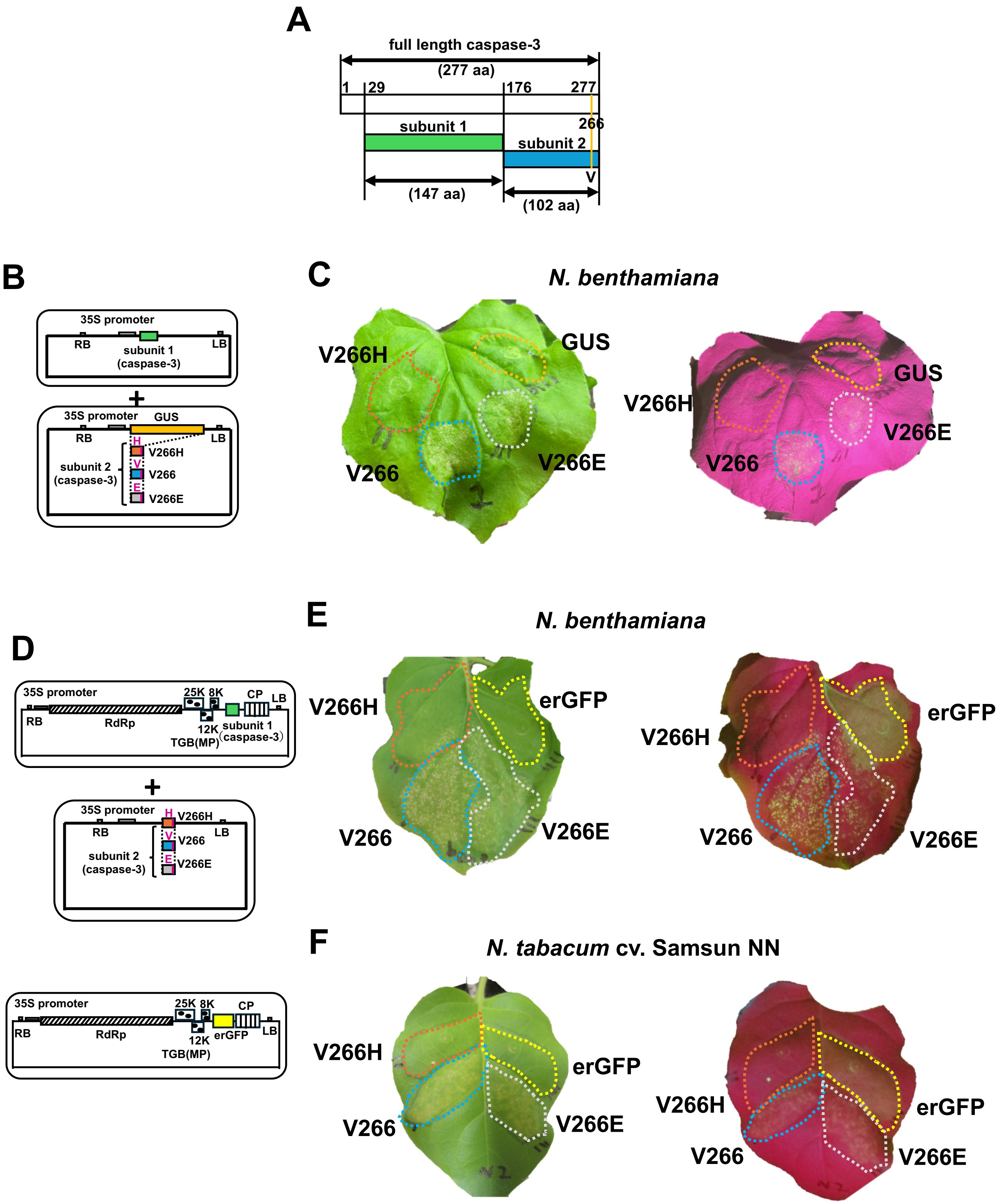

Supplementary Figure S3. Cell death induction by co-expression of genes encoding subunit 1 and subunit 2 of caspase-3.

(A) Schematic drawing showing the positions of each subunit of caspase-3. (B) Schematic drawing of the plasmids for the co-production of each subunit of caspase-3. The first plasmid encodes subunit 1 of

caspase-3 while the second one encodes the control GUS or subunit 2 of either wild type (V266)- or mutant type (V266H and V266E)-caspase-3. (C) *Agrobacterium* containing the plasmid encoding subunit 1 of caspase-3 was mixed with *Agrobacterium* containing the plasmid encoding one of three forms of subunit 2 of caspase-3 (V266H, V266, and V266E) or control GUS and co-infiltrated into the leaves of *N. benthamiana*. The infiltrated plants were kept at 25˚C. Turbidity of the *Agrobacterium* mixture was OD_{600} = 0.1 and the OD_{600} values corresponding to each *Agrobacterium* were both 0.05. Photographs were taken under white light (left) and blue light with a long-wave path filter (right) at 7 days after infiltration (dai). Dotted lines indicate each infiltrated area. (D) Schematic drawing of the plasmids for the co-production of each subunit of caspase-3. The first plasmid contains the infectious cDNA clone of the potato virus X (PVX) vector encoding subunit 1 of caspase-3, while the second one encodes subunit 2 of either wild type (V266)- or mutant type (V266H and V266E)-caspase-3. The third one encodes the infectious cDNA clone of the PVX vector encoding the control erGFP. (E), (F) *Agrobacterium* containing the plasmid encoding subunit 1 of caspase-3 was mixed with *Agrobacterium* containing the plasmid encoding one of three forms of subunit 2 of caspase-3 (V266H, V266, and V266E) and co-infiltrated into the leaves of (E) *N. benthamiana* and (F) *N.tabacum* cv. Samsun NN. The infiltrated plants were kept at (E) 25˚C or (F) 20˚C. Turbidity of the *Agrobacterium* mixture was OD_{600} = 0.4 and the OD_{600} values corresponding to each *Agrobacterium* were both 0.2. Photographs were taken under white light (left) and blue light with a long-wave path filter (right) at 12 dai. Dotted lines indicate each infiltrated area. As for the leaf region denoted as erGFP, only one kind of *Agrobacterium* (OD_{600} = 0.4) containing the infectious cDNA clone pf PVX vector encoding erGFP was used for infiltration.

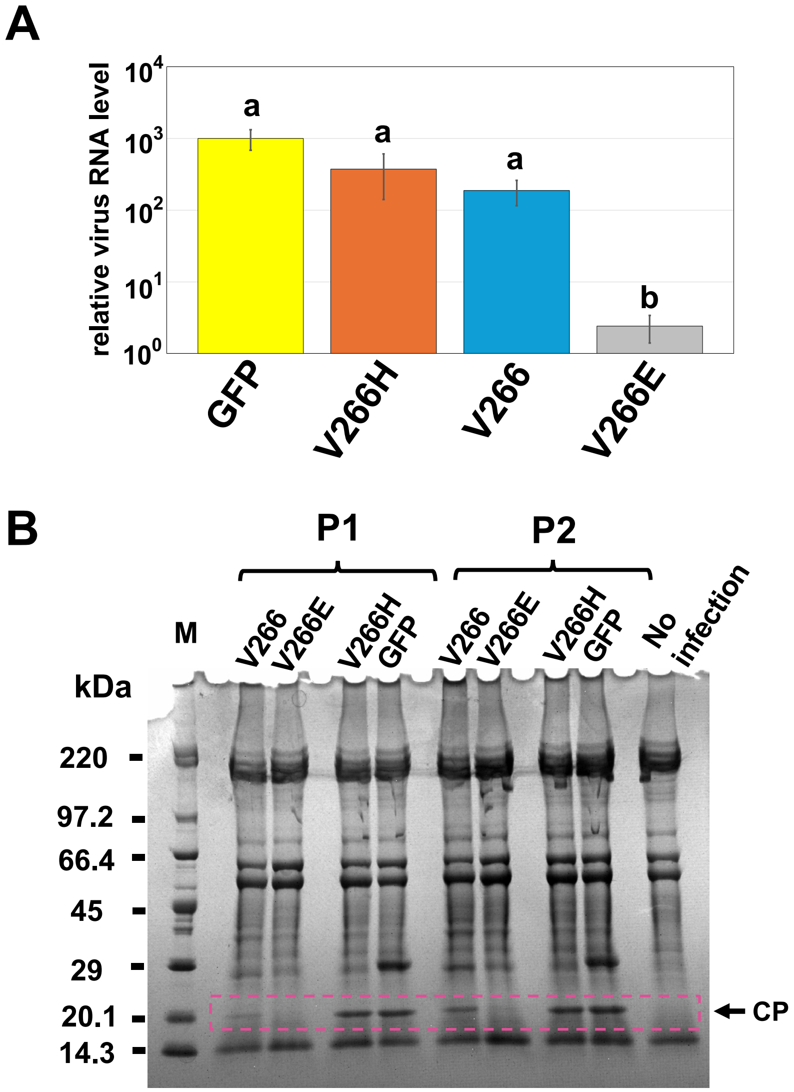

Supplementary Figure S4. Decreased accumulation of caspase-3-encoding ToMV in inoculated leaves.

(A) *Agrobacterium* containing the plasmid encoding GFP, V266H, V266 or V266E shown in Figure 1A were infiltrated into the leaves of *N. benthamiana* and the plants kept at 25˚C. Turbidity of the *Agrobacterium* in each infiltration solution was OD_{600} = 0.05. Total RNA was isolated from each region of the infiltrated leaves at 6 days after infiltration (dai), and the amount of virus RNA was then analyzed by RT-qPCR using the oligonucleotides for amplifying a part of the coding region for the virus RNA-dependent RNA polymerase, as shown in Supplementary Table S4. The relative virus RNA levels were calculated by setting the means of the GFP control to 10^3. The virus RNA levels were normalized against the *NbEF1α* mRNA levels. Values are mean ± SE (n = 5). Different letters above bars indicate statistically significant differences as analyzed by Steel-Dwass test ($p < 0.05$). (B) Cell extract was prepared using a 10-mM potassium phosphate buffer solution (pH 7.0) from each region of *N. benthamiana* leaves infiltrated with *Agrobacterium* containing the plasmid encoding GFP, V266H, V266, or V266E in Figure 1A at 6 dai and subjected to SDS-polyacrylamide gel electrophoresis with a gradient gel of 5 to 20%. Photographs of the gels stained with Coomassie Brilliant Blue are shown. The position of the CP band is indicated by an arrow, and the CP bands are enclosed in dotted rectangles. The notation above the gel (e.g., P1) indicates the individual plant used. The number following P is the individual number. Size marker lanes are indicated by the letter M. The size of each band is listed on the left side of the gel.

Supplementary Figure S5

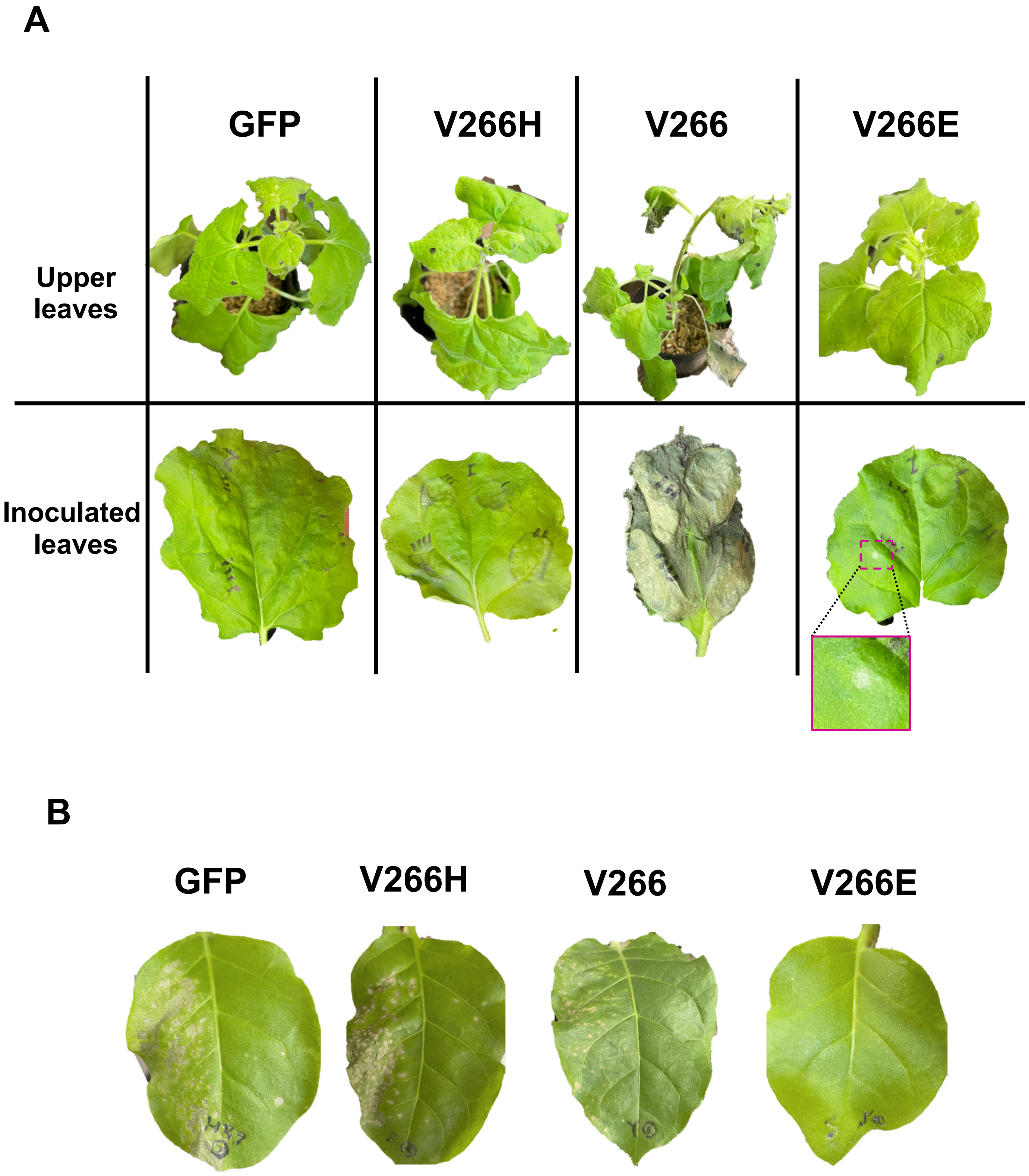

C

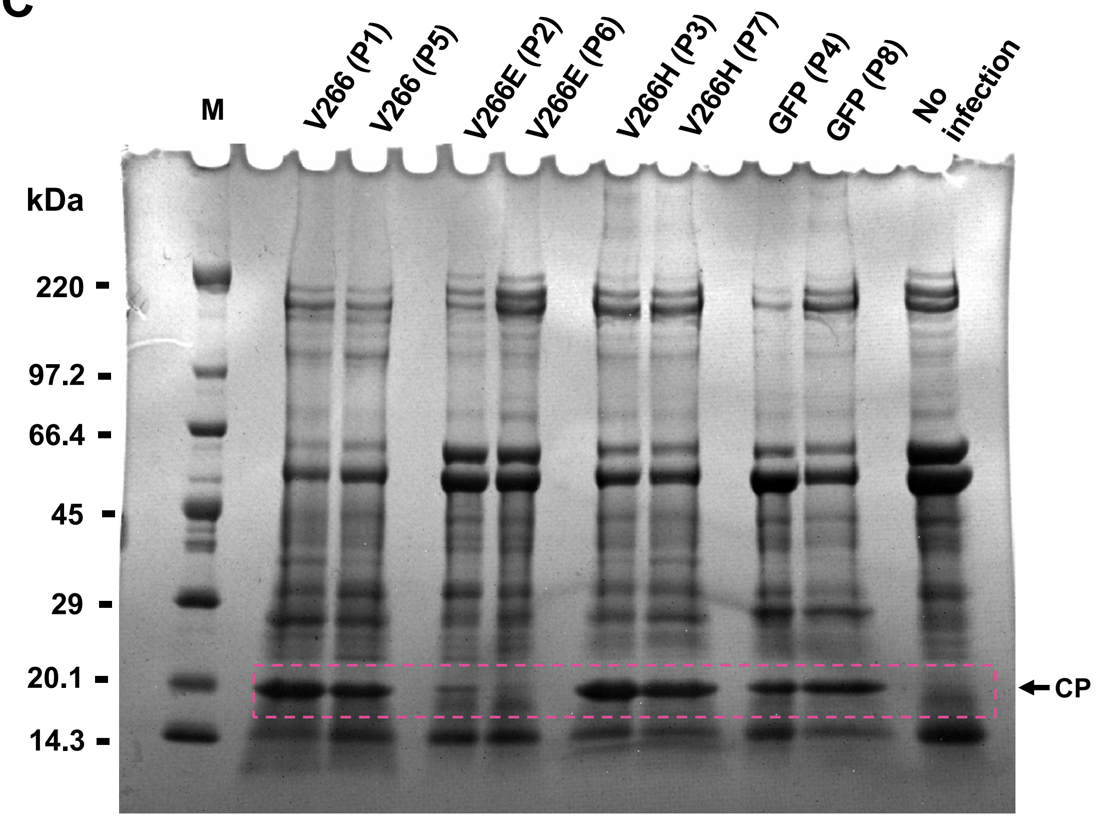

Supplementary Figure S5. Cell death symptoms and virus accumulation in non-inoculated upper leaves of *N. benthamiana* inoculated by caspase-3-encoding ToMV.

(A) *Agrobacterium* containing the plasmid encoding GFP, V266H, V266, or V266E shown in Figure 1A were infiltrated into the leaves of *N. benthamiana* and the plants kept at 25˚C. Turbidity of the *Agrobacterium* in each infiltration solution was OD_{600} = 0.01. Photographs were taken at 16 days after infiltration (dai) and show the entire plant body including upper leaves (top) and the inoculated leaf (bottom). An enlarged view of the photo section outlined in dotted squares is shown in the lower right (V266E). (B) Re-inoculation of leaf extracts. Cell extract was prepared from uninoculated upper leaves shown in (A) using a 50-mM potassium phosphate buffer solution (pH 7.0) at 16 dai. Then, the extract was mechanically inoculated onto the leaves of *N. tabacum* cv. Samsun NN, which contains the *N* gene, and the plants kept at 20˚C. Photographs were taken at 6 dai. (C) Cell extract prepared in (B) was subjected to SDS-polyacrylamide gel electrophoresis with a gradient gel of 5 to 20%. Photograph of the gel stained with Coomassie Brilliant Blue is shown. The position of the CP band is indicated by an arrow, and the CP bands are enclosed in a dotted rectangle. The notation in parentheses above the gel (e.g., P1) indicates the individual plant used. The number following P is the individual number. The lane for size marker is indicated by the letter M. The size of each band is listed on the left side of the gel.

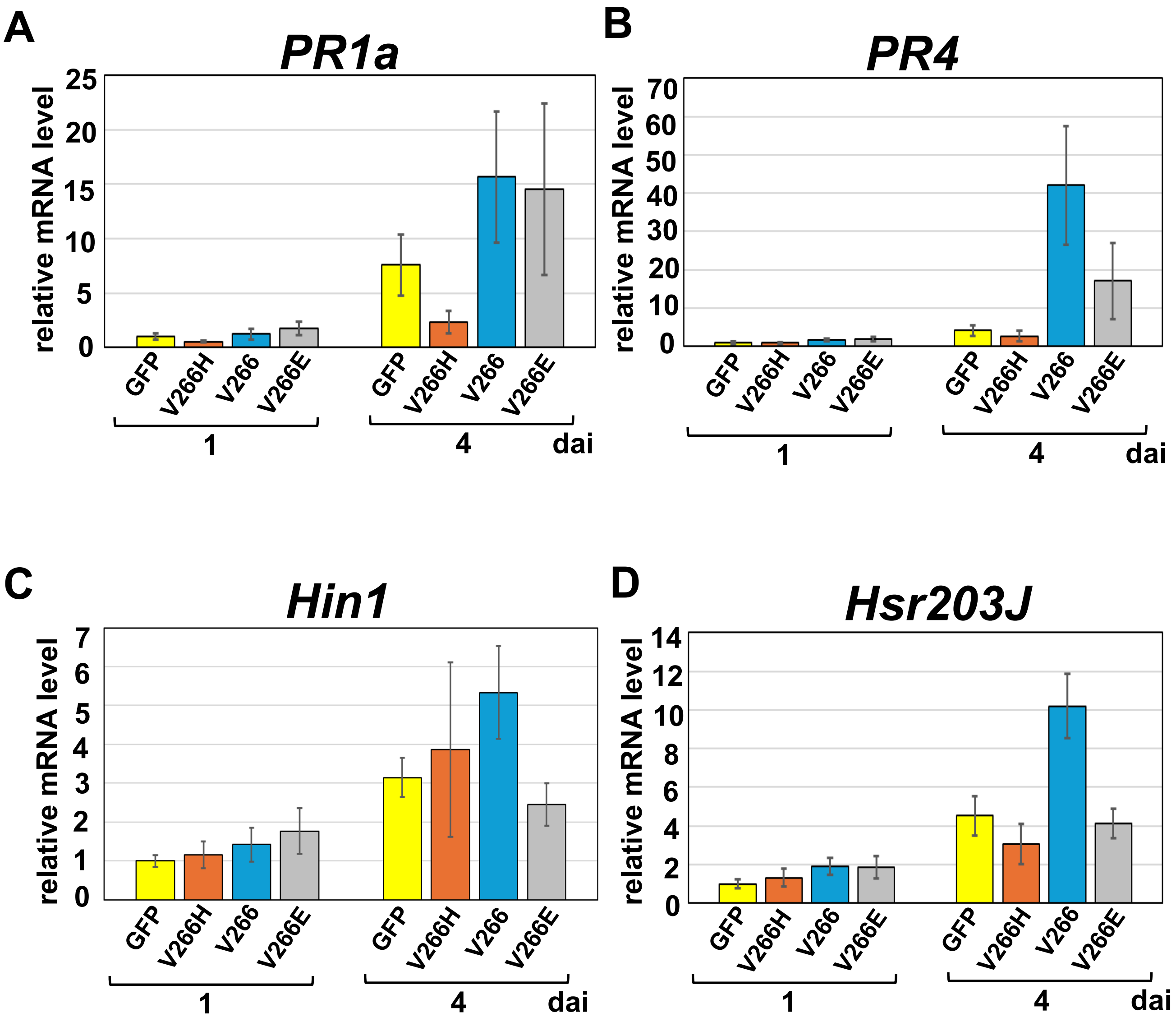

Supplementary Figure S6. Transcript levels of defense-related genes in the leaves expressing *caspase-3* by *Agrobacterium*-infiltration-type ToMV vector.
(A–D) *Agrobacterium* containing the plasmid encoding GFP, V266H, V266, or V266E shown in Figure 1A were infiltrated into the leaves of *N. benthamiana* and the plants kept at 25˚C. Turbidity of the *Agrobacterium* in each infiltration solution was OD_{600} = 0.3. Total RNA was isolated from each infiltrated region at 1 and 4 days after infiltration (dai), and the amount of virus RNA was then analyzed by RT-qPCR using oligonucleotides for the defense-related genes (A) *PR1a*, (B) *PR4*, (C) *Hin1*, and (D) *Hsr203J* of *N. benthamiana* (Supplementary Table S4) to evaluate the relative mRNA levels of each gene. The mRNA levels of these target genes were normalized to that of *NbActin* and the mRNA levels of the GFP control at 1 dai were fixed to 1 in each panel. Values are mean ± SE (n = 4). A nonparametric Steel-Dwass test was performed.

Supplementary Table S1. Oligonucleotides used for plasmid construction

oligonucleotide name	Sequence (5' to 3')
Cas3-Sub1-F01	TCTGGAATATCCCTGGACAAC
Cas3-Sub1-R01	GTCTGTCTCAATGCCACAGTCC
Cas3-Sub2-F02	AGTGGTGTTGATGATGACATGGC
Caspase3-human-F01	CACCATGGAGAACACTGAAAACTCAGTGG
Caspase3-human-R01	GTGATAAAAATAGAGTTCTTTTGTGAGCATGG
Caspase3-human-R02	TTAGTGATAAAAATAGAGTTCTTTTGTGAGCATGG
Caspase3-human-R03	GTGATAAAAATAGAGTTCTTTTGTGAGCATGGATTCAATACATGGAATCTGTTTCTTTGC
Caspase3-human-R04	TTAGTGATAAAAATAGAGTTCTTTTGTGAGCATGGAATGAATACATGGAATCTGTTTCTTTGC
hCas3-pGR107-F01	CTAGCATCGATTCCCGGGCACCATGGAGAACACTGAAAACTC
hCas3-pGR107-R01	ATCGGCGGTCGACCCGGGTTAGTGATAAAAATAGAGTTCTTTTGTG
hCas3Sub1-pGR107-F01	CTAGCATCGATTCCCGGGCACCATGTCTGGAATATCCCTG
hCas3Sub1-pGR107-R01	ATCGGCGGTCGACCCGGGTTAGTCTGTCTCAATGCCACAG
pENTR-F01	AAGGGTGGGCGCGCCGACCCAGC
pENTR-F02	TAAAAGGGTGGGCGCGCCGACCCAGC
pENTR-R02	CATGGTGAAGGGGGCGGCCGCGG
TogJ-pGLW3-R01	GAAATTCGGGGGATCCGTCGACGCGTGG
TogJ-R04	ACCATGATTACGAATTCCCGG
TogJ-seq-F10-2	GATAATTTGATTGAAGATGAAG
XV-F01	GTGCGGAAATCGCGCAGCGTTTGG
XV-pGR107 F01	CTAGCATCGATTCCCGGGCACCATGAAAGCGTTAAC
XV-pGR107-R02	ACCGTTCATCGGCGGTCGACCCGGGTTAATCCCCACCGTAC
XV-R01	GTGTCGGCGGCATACCTGTCTGG
XVE-F01	CACCATGAAAGCGTTAACGGCCAGGC
XVE-R02-w/o-stop-codon	GACTGTGGCAGGGAAACCCTCTGCC
XVE-V-R01	ATCCCCACCGTACTCGTCAATTCC

Supplementary Table S2. Plasmids used in this study

1. Plasmids for gene expression

plasmid name	plasmid ID	type of caspase-3 or name of control gene	type of vector plasmid	Reference
pART27-35Sa-GUS	ACEK11	*GUS*	Agrobacterium-infiltration-type plasmid for direct gene expression by the CaMV 35S promoter	Suzuki et al. 2024
pART27-35Sa-Cas3	ACCO1	full length		this work
pART27-35Sa-Cas3-V266E	ACCP2			
pART27-35Sa-Cas3-V266H	ACCQ4			
pART27-35Sa-Cas3-sub1	ACCE9	subunit 1		
pART27-35Sa-Cas3-sub2	ACBJ2	subunit 2		
pART27-35Sa-Cas3-sub2-V266E	ACBQ3			
pART27-35Sa-Cas3-sub2-V266H	ACBU1			
pGL-TocJ-GFP	ACHR7	*GFP*	*Agrobacterium* -infiltration-type ToMV vector plasmid	this work
pGL-TogJ-Cas3	ACIY1	full length		
pGL-TogJ-Cas3-V266E	ACIS1			
pGL-TogJ-Cas3-V266H	ACIZ2			
pGR107-erGFP	DRZ19	*erGFP*	Agrobacterium-infiltration-type PVX vector plasmid	Suzuki et al. 2024
pGR107-Cas3	ACDL5	full length		this work
pGR107-Cas3-V266E	ACDI3			
pGR107-Cas3-V266H	ACDN2			
pGR107-Cas3-sub1	ACDS4	subunit 1		
pTocJ-GFP	-	*GFP*	mechanical inoculation-type ToMV vector plasmid	Hori and Watanabe 2003
pTogJ-Cas3	ABTM20	full length		this work
pTogJ-Cas3-V266E	ABWB9			
pTogJ-Cas3V-266H	ABWS2			

2. Plasmids used during the construction of gene expression plasmids

plasmid name	plasmid ID	type of caspase-3 or the gene of interest	type of plasmid or where used	Reference
pGEM-Cas3	ABSR4	full length	Cloning of human *caspase-3* gene	this work
pENTR-Cas3-V266E_no_stop_codon	ABVD1		Construction of entry plasmids	
pENTR-Cas3	ABTE7		entry plasmids	
pENTR-Cas3-V266E	ABVW5			
pENTR-Cas3-V266H	ABWO2			
pENTR-Cas3-sub1_with_stop_codon_first_stage	ACAD3	subunit 1	Construction of entry plasmids	
pENTR-Cas3-sub1_with_stop_codon	ACCB1		entry plasmids	
pENTR-Cas3-sub2_with_stop_codon	ACAH1	subunit 2	entry plasmids	
pENTR-Cas3-sub2-V266E_with_stop_codon	ACBC1			
pENTR-Cas3-sub2-V266H_with_stop_codon	ACBE1			
pART27-35Sa-GWB-DHA	DAY5	-	vector plasmid	Ogata et al. 2012
pTogJ	AADE8	-		Hori and Watanabe 2003
pGLW3	-	-		Sasaki et al. 2013
pMDC150	-	*XVE*	Construction of modified PVX vector	Brand et al. 2006
pENTR-XVE_no_stop_codon	ABQY2	*XVE*		this work
pENTR-XV_with_TAA_stop_codon	ABYP2	*XV*		
pENTR-XV_no_Mlu I site_with_TAA_stop_codon	ACAJ3	*XV*		
pTogJ-XV_no_MluI_site_with_TAA_stop_codon	ACBY1	*XV*		
pGR107-XV_no_MluI_site_with_TAA_stop_codon	ACCX1	*XV*		

References

Brand L, Hörler M, Nüesch E, Vassalli S, Barrell P, Yang W, Jefferson RA, Grossniklaus U, Curtis MD (2006) A versatile and reliable two-component system for tissue-specific gene induction in Arabidopsis. Plant Physiol 141: 1194–1204

Hori K, Watanabe Y (2003) Construction of a tobamovirus vector that can systemically spread and express foreign gene products in solanaceous plants. Plant Biotechnol 20:129–136

Ogata T, Kida Y, Arai T, Kishi Y, Manago Y, Murai M, Matsushita Y (2012) Overexpression of tobacco ethylene response factor NtERF3 gene and its homologues from tobacco and rice induces hypersensitive response-like cell death in tobacco. J Gen Plant Pathol 78: 8–17

Sasaki N, Takaoka M, Sasaki S, Hirai K, Meshi T, Nyunoya H (2013) The splice variant Ntr encoded by the tobacco resistance gene N has a role for negative regulation of antiviral defense responses. Physiol Mol Plant Pathol 84: 92–98

Suzuki H, Ito T, Ogata T, Tsukahara Y, Nelson RS, Sasaki N, Matsushita Y (2024) Overexpression of NtERF5, belonging to the ethylene response factor gene family, inhibits potato virus X infection and enhances expression of jasmonic acid/ethylene signaling marker genes in tobacco. J Gen Plant Pathol 90: 125–133

Supplementary Table S3. Turbidity of bacteria used for *Agrobacterium*-infiltration

	plasmid name	plasmid ID	Turbidity of bacteria OD_{600} each	Turbidity of bacteria OD_{600} total
Figure 1	pGL-TogJ-Cas3	ACIY1	0.3	0.3
	pGL-TogJ-Cas3-V266E	ACIS1		
	pGL-TogJ-Cas3-V266H	ACIZ2		
	pGL-TocJ-GFP	ACHR7		
Figure 2	pART27-35Sa-Cas3-sub1	ACCE9	0.05	0.18
	pART27-35Sa-Cas3-sub2	ACBJ2	0.05	
	pART27-35Sa-Cas3-sub2-V266E	ACBQ3		
	pART27-35Sa-Cas3-sub2-V266H	ACBU1		
	pART27-35Sa-GUS	ACEK11		
	pGL-TocJ-GFP	ACHR7	0.08	
Figure 3A	pART27-35Sa-Cas3-sub1	ACCE9	0.1	0.2
	pART27-35Sa-Cas3-sub2	ACBJ2	0.1	
	pART27-35Sa-Cas3-sub2-V266E	ACBQ3		
	pART27-35Sa-Cas3-sub2-V266H	ACBU1		
	pART27-35Sa-GUS	ACEK11		
Figure 3B	pART27-35Sa-Cas3-su1	ACCE9	0.05	0.18
	pART27-35Sa-Cas3-sub2	ACBJ2	0.05	
	pART27-35Sa-Cas3-sub2-V266E	ACBQ3		
	pART27-35Sa-Cas3-sub2-V266H	ACBU1		
	pART27-35Sa-GUS	ACEK11		
	pGL-TocJ-GFP	ACHR7	0.08	
Supplementary Figure S1	pGR107-Cas3	ACDL5	0.01	0.01
	pGR107-Cas3-V266E	ACDI3		
	pGR107-Cas3-V266H	ACDN2		
	pGR107-erGFP	DRZ19		
Supplementary Figure S3C	pART27-35Sa-Cas3-sub1	ACCE9	0.05	0.1
	pART27-35Sa-Cas3-sub2	ACBJ2	0.05	
	pART27-35Sa-Cas3-sub2-V266E	ACBQ3		
	pART27-35Sa-Cas3-sub2-V266H	ACBU1		
	pART27-35Sa-GUS	ACEK11		
Supplementary Figure S3E	pGR107-Cas3-sub1	ACDS4	0.2	0.4
	pART27-35Sa-Cas3-sub2	ACBJ2	0.2	
	pART27-35Sa-Cas3-sub2-V266E	ACBQ3		
	pART27-35Sa-Cas3-sub2-V266H	ACBU1		
	pGR107-erGFP	DRZ19	0.4	0.4
Supplementary Figure S3F	pGR107-Cas3-sub1	ACDS4	0.2	0.4
	pART27-35Sa-Cas3-sub2	ACBJ2	0.2	
	pART27-35Sa-Cas3-sub2-V266E	ACBQ3		
	pART27-35Sa-Cas3-sub2-V266H	ACBU1		
	pGR107-erGFP	DRZ19	0.4	0.4
Supplementary Figure S4	pGL-TogJ-Cas3	ACIY1	0.05	0.05
	pGL-TogJ-Cas3-V266E	ACIS1		
	pGL-TogJ-Cas3-V266H	ACIZ2		
	pGL-TocJ-GFP	ACHR7		
Supplementary Figure S5	pGL-TogJ-Cas3	ACIY1	0.01	0.01
	pGL-TogJ-Cas3-V266E	ACIS1		
	pGL-TogJ-Cas3-V266H	ACIZ2		
	pGL-TocJ-GFP	ACHR7		
Supplementary Figure S6	pGL-TogJ-Cas3	ACIY1	0.3	0.3
	pGL-TogJ-Cas3-V266E	ACIS1		
	pGL-TogJ-Cas3-V266H	ACIZ2		
	pGL-TocJ-GFP	ACHR7		

Supplementary Table S4. Oligonucleotides used in RT-qPCR analysis

target gene	Oligonucleotide name	Sequence (5'-3')	Amplification length(bp)	Source of target gene nucleotide sequence information
ToMV RdRp	TogJ-F13	ATATACCTGCCGACGAGTTC	110	X02144
	TogJ-R13	GTTGACGTGTGAATCTTCGAG		
NbActin	NbActin-F1	CAGCTCATCCGTGGAGAAGA	279	AY179605
	NbActin-R1	AGGATACGGGGAGCTAATGC		
NbEF1α	NbEF1a-F1	AGAGGCCCTCAGACAAAC	132	AY206004
	NbEF1a-R1	TAGGTCCAAAGGTCACAA		
NbPR1a	acidicPR1-F11	TAGTCATGGGATTTGTTCTC	152	>Niben261Chr13-5460301-5461900
	acidicPR1-R11	ACCTACATCTGCACGAGCTG		
NbPR4 (*PR-4B*)	Nb_PR4_F(EH365959)	CAGAACATTAACTGGGATTTGAGAG	189	EH365959
	Nb_PR4_R(EH365959)	CACTGTTGTTTGAGTTCCTGTTC		
NbHin1	Hin1_real_F2	GCGTATTAGGCTTAAGGTTGG	142	>Niben261Chr01-148349701-148351400
	Hin1_real_R1b	CTACCAATCAAAATGGCATCTG		
NbHsr203J	Hsr203-real-F2b	TAACAATGGAGTGGGACATAGC	109	>Niben261Chr15-97954735-97955742
	Hsr203-real-R1	GATGAACTCTGCAACGGCTTC		

Oligonucleotides to each gene were designed based on the sequence information obtained from the International Nucleotide Sequence Database (INSD; GenBank/EMBL/DDBJ) or Nicotiana benthamiana Genome V2.6.1 BLAST dataset (https://solgenomics.net/organism/Nicotiana_benthamiana/genome). For *ToMV RdRp*, *NbActin*, *NbEF1α* and *NbPR4* genes, INSD accession numbers are shown. For the rest, the information including chromosome number and its nucleotide sequence potions is shown (eg. In the case of >Niben261Chr13-5460301-5461900 of the *NbPR1a*, Chr13 means chromosome number and 5460301-5461900 means nucleotide potions.)
